# A Plastic Temporal Brain Code for Conscious State Generation

**DOI:** 10.1155/2009/482696

**Published:** 2009-07-22

**Authors:** Birgitta Dresp-Langley, Jean Durup

**Affiliations:** ^1^Centre National de la Recherche Scientifique (CNRS - UMR 5508), Université Montpellier 2, CC048 34095 Montpellier Cedex 5, France; ^2^16 rue Romain Rolland, 34200 Sète, France

## Abstract

Consciousness is known to be limited in processing capacity and often described in terms of a unique processing stream across a single dimension: time. In this paper, we discuss a purely temporal pattern code, functionally decoupled from spatial signals, for conscious state generation in the brain. Arguments in favour of such a code include Dehaene et al.'s long-distance reverberation postulate, Ramachandran's remapping hypothesis, evidence for a temporal coherence index and coincidence detectors, and Grossberg's Adaptive Resonance Theory. A time-bin resonance model is developed, where temporal signatures of conscious states are generated on the basis of signal reverberation across large distances in highly plastic neural circuits. The temporal signatures are delivered by neural activity patterns which, beyond a certain statistical threshold, activate, maintain, and terminate a conscious brain state like a bar code would activate, maintain, or inactivate the electronic locks of a safe. Such temporal resonance would reflect a higher level of neural processing, independent from sensorial or perceptual brain mechanisms.

## 1. Introduction

In the last twenty years, consciousness studies have produced a considerable bulk of theoretical and experimental works concerned with trying to answer two critical questions: (1) is a scientifically operational definition of consciousness possible and (2) where and how is this phenomenon produced in the brain? Being able to answer the second question entirely depends on whether a valid answer to the first one can be given. Looking back on the various different approaches in this field (e.g., [1–9]), neuroscientists are left with the conclusion that a fully operational yet comprehensive definition of the phenomenon still poses a fundamental problem, as pointed out in one of the more recent theoretical papers by Block [10], where the author argues for an “abstract solution” to the “problem of consciousness.” Given that phenomenal consciousness by far exceeds cognitive accessibility and performance, its neural basis is not to be identified with the neural basis of any particular cognitive process taking place within consciousness, as Block [10] and others have argued. The “hard problem of consciousness” (e.g., [11–13]) or the seemingly insurmountable difficulty to explain through which mechanisms the conscious *I* which is experienced in terms of *I do*, *I feel,* or *I am* arises in the brain has, indeed, remained an unresolved issue. In this paper, we propose an abstract solution to this problem by suggesting a biophysical model which dissociates the particular cognitive processes which may take place within consciousness, such as conscious perception, for example, from the neural mechanisms that trigger, maintain, and terminate a conscious state (*I do*, *I feel*, *I am*) in the brain. The functional assumptions of our model clarify how such a particular brain state may arise from purely temporal resonance of memory signals in neural circuits, and how this may happen in the absence of any stimulus input, attention, or perception associated with a specific conscious behaviour.

### 1.1. Conscious Perception and Behaviour the Limiting Factor

Theories of consciousness based on cognitive performance or conscious (as opposed to non-conscious) perception do not address the “hard problem of consciousness.” The science of consciousness has thus far been reduced to looking for measures and neural correlates of conscious behaviour that reflects particular cognitive processes. These measures and correlates (see, e.g., [14], for a review) are nothing more than partial traces, found in specific behaviour like guided attention, active conscious perception and report, or conscious memory recall, of a far more complex and intricate phenomenon. Myriad studies in which a particular behaviour is investigated to understand consciousness have been reported. Dehaene et al. [9], for example, approached consciousness in terms of conscious report. These authors suggest that a human subject is phenomenally conscious when some critical event is reliably reported and argue that consciousness may, therefore, be defined in terms of “access of information to conscious report.” Interestingly, such a restriction of phenomenal consciousness to processes that enable information to access a certain level of conscious representation is grounded in Block's earlier theory of what he called “access consciousness” [5]. However, considering conscious report of human observers as an indicator for mechanisms which give access to consciousness leads to several critical questions, which remain to be answered. Does information made accessible to conscious report correspond to ongoing or past, to real or imagined events? Does the conscious experience that is subject to conscious report occur well before, immediately before, or during the report? How long would the experience be expected to last afterwards? In short, is studying conscious perception and attention sufficient to understand the mechanisms that produce consciousness in the brain? 

 The logic of scientific explanation requires that the nature of the *explanandum*, or what is to be explained, is adequately derived from the *explanans*, or explanation given. Considering the case of studies focussed on conscious perception, we have to bear in mind that any specific consciously performed behaviour is no more than a particular expression of the *explanandum * (consciousness). It neither occurs consistently nor systematically whenever the individual is conscious. An *explanans* derived from such a particular form of expression is adequate only with regard to the specific perceptual process studied, not with regard to the *explanandum * (consciousness) as such. Specific behaviour of conscious perception and conscious report and memory recall has been correlated with specific neural activities in the occipital and the late parieto-frontal regions of the brain (see [15, 16] or [9] for extensive reviews and discussions). Along the same line of reasoning, these brain activities may be interpreted adequately in terms of correlates of a particular process of conscious behaviour highlighted by the experimental data, but not in terms of the neural correlates of consciousness as such. 

 Studies of behaviour which reflects what appear to be transitions between nonconscious and conscious processes such as change blindness (e.g., [17]) have given rise to interpretations of conscious perception in terms of a selective process which opens access to higher levels of information processing. In change blindness, human observers are unable to detect important changes in briefly presented visual scenes disrupted by blinks, flashes, or other visual masks just before the changes occur. This phenomenon may be seen as a particular kind of preconscious perception ([1, 18]). In fact, what happens in change blindness is that observers fail to report what they actually see because they believe that what is there is what they have seen just before. Such belief blocks the selective process which would otherwise enable the new information contained in the new visual scene to access conscious perception. Change blindness has been considered to result from top-down inhibition of ongoing stimuli (cf. [18]), preventing their conscious perception. Change blindness phenomena are particular cases where the conscious state is filled with a dominant memory representation of a previously experienced event. This suggests that there is a selective brain mechanism that makes information accessible to consciousness. More importantly, such a selective mechanism may explain how conscious states are generated in the complete absence of awareness and perception, as in lucid dreaming, for example.

### 1.2. Lucid Dreaming and Hallucinations: Conscious Experience without Perception

As pointed out already more than a century ago by William James [11], consciousness encompasses far more than being wakeful and able to consciously perceive and remember events which occur or have occurred in the world. When we dream intensely, we are not attentive to *stimuli*, but we are phenomenally conscious. We may even be able to access and report these phenomenal data several hours later, when we recount our dreams over breakfast. Similarly, patients suffering from mental disorders such as schizophrenia experience hallucinatory events consciously in the absence of external stimuli which trigger the experience. How hallucinations may arise in the brain from hyper-activation of volitional signals, triggering fully conscious and often vivid visual imagery and internally generated “voices” in hallucinating patients, has been discussed extensively by [19] on the basis of his Adaptive Resonance Theory (ART), to which we will get back later. 

 Baars (e.g., [20]) referred to phenomenal consciousness as the “theatre of the mind,” which is reminiscent of writings from the first book (Part 4, Section 6) of the *Treatise of Human Nature* (1740) in which the Scottish Philosopher David Hume compared phenomenal consciousness to a theatre with a scene of complex events where various different sensations make their successive appearance in the course of time:

“The mind is a kind of theatre, where several perceptions successively make their appearance; pass, repass, glide away, and mingle in an infinite variety of postures and sensations. There is properly neither simplicity in it at one time, nor identity in different, whatever natural propension we may have to imagine that simplicity and identity. The comparison of the theatre must not mislead us. They are the successive perceptions only, that constitute the mind; nor have we the most distant notion of the places where these scenes are represented, or of the materials of which it is composed.”

Hume's phenomenal description defines consciousness in terms of successive moments in time where feelings and sensations, not necessarily related to ongoing external events or *stimuli*, appear and vanish from the mind. 

 LaBerge et al. ([21]) argue that dreaming of perceiving and doing is equivalent to perceiving and doing. Such a view is supported by evidence for a functional equivalence of psycho-physiological correlates of consciousness in active wakeful observers and during lucid dreaming, which occurs in REM sleep phases. Lucid dreaming and equivalent wakeful activities are measured in terms of relatively short EEG signal epochs indicating a specific activation level of the central nervous system (e.g., [22]). In addition, it has been shown (e.g., [23]) that the invariant patterns of change in quantitative EEG analysis during anaesthesia and wakefulness are reliable brain correlates of what we will refer to here as the conscious brain state.

### 1.3. The Conscious Brain State

The notion of the conscious state was discussed earlier by Tononi & Edelman [24] and Edelman [25], based on a definition proposed by von der Malsburg [26] in terms of a continuous brain process with a limited duration. Such an abstract definition of consciousness allows separating certain properties of the physiological state of the brain during a conscious experience from the subjective phenomenal contents that are being experienced. Moreover, it satisfies a major constraint to the scientific study of consciousness, the so-called law of parsimony (*lex parsimoniae*). The latter is both ethically and pragmatically grounded in the philosophy of science of the English cleric William of Occam (14th century: *“entia non sunt multiplicanda necessitatem”*) and states that the explanation of a phenomenon should resort to as few “entities” (mechanisms, processes, laws) as possible. 

 We argue that the definition of a conscious state of the brain, in which *I am*, *I do,* or *I feel*, most adequately defines a scientifically operational *explanandum*. This latter is then to be accounted for by an *explanans* in terms of the fewest mechanisms needed for its generation. Conscious states are neither identical nor reducible to states of awareness or vigilance (see also [27]). Particular cognitive processes such as conscious memory recall, attention, conscious perception, and volition ([9, 19, 28–31]) may or may not be part of the expression of a conscious state at a given moment in time. A conscious state is a specific functional state of the brain, one that enables conscious experience of various subjective contents but is functionally independent from these subjective contents. In a conscious state where I feel that *I am tired*, for example, the brain substrate for the conscious nature of this feeling is functionally independent from the brain signals produced by my physiological and psychological states (*tired*) at that given moment. How the temporal signatures that generate conscious states become progressively independent from brain signals involved in particular sensorial and perceptual processes in the course of development will be discussed later. 

 John [32] argued that the most probable invariant level of neural activity or coherent functional interaction among brain regions that can be measured when a person is in a conscious state is the best possible approximation to what he called the “conscious ground state of the brain.” The conscious ground state of the brain results from specific activities in neural circuits with no more than two (see also [25]) general functional characteristics: (1) very limited information processing capacity (see, e.g., [33–35]) and (2) a unique representational content for a limited and relatively short duration (e.g., [8, 36–38]). The database from which a conscious state draws this representational content is steadily updated through nonconscious processes, which constitute by far the largest part of all brain activity (e.g., [39–41]). Conscious information processing involves very little of such activity. It has been argued that this functional constraint is the limiting factor to any theory of consciousness ([25, 42–44]). Conscious information processing relies on serial processing and allows for only a limited amount of information to be dealt with in the time span of a given conscious state. This is reflected, for example, by the fact that most people cannot consciously follow two ideas at the same time, or consciously execute two tasks simultaneously (e.g., [45, 46]). Thus, it seems quite clear that the conscious brain state relies entirely on working memory capacity ([47–50]).

## 2. Time-Bin Model for Conscious State Generation in the Brain

The model we propose here is based on the idea that a conscious brain state is generated on the basis of purely temporal coincidences of memory signals, sometimes called representations, defined as by Churchland ([51, Page 64]) in terms of “patterns of activity across groups of neurons which carry information.” Such patterns of neural activity are described by unique signal sequences across time. These constitute the potential temporal signatures of conscious states. 

### 2.1. The Temporal Signatures of Conscious Brain States

Earlier models based on the functional properties of working memory have attempted to clarify how groups of neurons could produce a specific temporal signal sequence, or temporal signature, that is sufficient to activate, maintain, and terminate a conscious state in the brain. Such a temporal signature would fulfil a double function: it would enable the generation of specific conscious brain states that are well distinguished from non-conscious brain states, and it would provide ready accounts for both their selective nature and the fact that they may occur in the mind more than once. A certain class of theoretical approaches to working memory, such as the Lisman-Idiart-Jensen memory model ([52–57]) has proposed temporal mechanisms based on some of the empirical findings summarized above, postulating a working memory with a maximum processing capacity of only a few items, where each such item is represented by the firing activity of a cell assembly, the so-called coding assembly, in a well-defined temporal window. Specific numerical predictions were developed on the basis of such memory models (for details, see [56, 58]). Başar [59] and Başar et al. [60] considered cognitive transfer activities to be based on oscillations at specific temporal frequencies, combined like the letters of an alphabet to deliver a temporal code for conscious brain activity measurable through wavelet analysis of EEG or event-related potentials (ERPs). While these numerical models illustrate both the plausibility and the potential power of temporal codes in the brain, there is a major difference between such models and the one we propose here to account for conscious state generation. Our hypothesis relies on particular dynamics of temporal messages, or resonant time-bin messages, produced by a complex system (the brain) within massively distributed circuits of neurons. It implies that these temporal dynamics are oscillatory, since all known resonance mechanisms are by their physical nature oscillatory, but does not make predictions regarding any particular frequency bands. Recent models' simulations have invoked possible cortical constraints for the genesis of particular conscious perceptual events, requiring a synchronization of oscillations in visual cortical and prefrontal areas ([61]) for example. Our own model postulates a functional separation between perception related neural activities in functionally identified cortical regions and the temporal neural activity patterns which generate conscious states and reflect a higher processing level. Synchronization of neural activities generated in the different functionally identified areas of the brain is not required to enable such processing. 

 Taking the general idea of temporal codes in the brain further, we argue that unique combinations of temporal sequences beyond some critical activity threshold generate unique conscious states, which may be regenerated whenever that signature is retrieved again, either by the same set of neurons or any other set capable of producing it. Such neural timing for conscious state generation would rely on simultaneous *supra* threshold activation of sets of cells within dedicated neural circuits in various arbitrarily but not necessarily randomly determined *loci* of the brain. The intrinsic topology that determines which single cell of a given circuit produces which spike pattern of a given temporal signature is, therefore, independent of the topological functional organization of the brain. 

 This assumption that a conscious brain state is triggered by temporal signals of neural circuits that operate at a higher level and independently from other functionally specific circuitry suggests a way of thinking that is radically different from that offered by most current approaches. Such functional independence has the considerable advantage that, should subsets of coding cells be destroyed, other subsets could still deliver the temporal code for conscious states elsewhere in the brain. This hypothesis is fully justified in the light of evidence for a considerable plasticity of functional brain organization (e.g., [62]), which we will discuss later herein in greater detail.

### 2.2. Temporal Limits of the Conscious Brain State

Just as the temporal signal sequence or activity pattern of any single coding cell is determined by its firing activity across a certain length of time, the temporal signature of a conscious state is also linked to duration, with variations in the limited dynamic range of a few hundreds of milliseconds. These temporal limitations have led many authors to link a conscious brain state to a specific conscious experience, or “psychological moment” ([24, 63, 64]) the particular expressions of which have been investigated in neurobiological and psychophysical studies (e.g., [64–81]). Neural network simulations matching the psychophysical and neurobiological data have been proposed ([31, 82]). 

 Here, for important theoretical reasons stated in the introduction, we attempt to go beyond explanations which link the conscious state to any specific conscious content or experience, bearing in mind that our model is to suggest an “abstract solution” to the problem of consciousness (cf. [10]). Such an abstract solution could be, we argue, a purely temporal pattern code underlying the genesis of conscious states in the brain. To decipher such a code in neural signal patterns, the biophysical duration *t* of a conscious state may be divided into time bins [83], the duration of which is limited by the accuracy of neuronal timing, or the lower limit of biophysics. Each such bin is expressed through the parameter Δ*t*, which represents the sum of standard deviations for the time delay of synaptic transmission including the duration of the refractory period. An average estimate of 6 milliseconds for Δ*t* appears reasonable in the light of currently available data, which give estimates between 3 and 10 ms for this parameter ([84–89]). Interspike intervals and integration times of cortical neurons display a similar dynamic range [90]. Under the simple assumption that within each time bin there is either a signal or no signal, which is derived from McCullough & Pitts' [91] germinal work on information transmission in neural networks, the information content of a bin with a signal is 1 bit. On the basis of an average duration *t * of 300 milliseconds for the conscious state, a Δ*t* of 6 milliseconds for each bin, the information content of a conscious brain state with average duration would not exceed 300/6 = 50 bits. A similar computation of the maximum quantity of information conveyed by a duration *t* with a number of temporal windows identified by a given Δ*t* was proposed by MacKay & McCulloch [92]. Other time-based models of biophysical information processing related to conscious brain states were suggested later by Thorpe et al. [93] and VanRullen et al. [94]. Approaches in terms of dynamic analyses of correlated oscillations in cortical areas at various frequencies (e.g., [95]) and functional interactions between gamma and theta oscillations in different structures of the brain (e.g., [96]) are consistent with biophysical time estimates given previously. How an immense variety of neural signals would be processed to generate a purely temporal code for conscious brain states becomes clearer in the light of functional properties of reverberant neural circuits in the brain, and the concept of a functional separation between spatial and temporal neural messages in the course of long-distance signal propagation.

### 2.3. Long-Distance Signal Propagation and Functional Segregation of Signal Contents

Reverberant circuits or loops in the brain have their own intrinsic toplogy (e.g. [9, 31, 97–101]). Reverberant neural activity has been found in thalamo-cortical ([102–104]) as well as in cortico-cortical pathways ([105–108]). Reverberant neural activity as such is a purely temporal process that generates feed-back loops in the brain, referred to by some as “re-entrant circuits” ([24, 25, 98, 109–117]). Reverberation is an important functional property of the brain because without it, the conscious execution of focussed action would be difficult, if not impossible [108]. 

 Dehaene et al. [9] suggested that consciousness relies on the extension of local brain activation to higher association cortices that are interconnected by long-distance connections forming reverberating neuronal circuits extending across distant perceptual areas. We believe that the major functional advantage of such long-distance reverberation could be that it allows holding information online for durations that are unrelated to the duration of a given stimulus and long enough to enable the rapid propagation of information through different brain systems. Functional imaging studies have associated conscious brain activity with the parieto-frontal pathways, others suggested occipital correlates (see [16], for an extensive review). What both these brain regions have in common, interestingly, is that they are protected from fluctuations in sensory signals and therefore allow information sharing across a broad variety of higher cognitive processes, well beyond sensory perception. 

 We argue that such selective information sharing and reduction of signal variations would provide an important functional advantage to the systems in the brain which produce the conscious state code, because at such a stage of processing, such systems would be no longer required to sort out highly complex cross-talk between signals from a multitude of different channels. Thus, the major functional hypothesis of our model claims that long-distance reverberation of neural signals across long-range connections enables functional segregation of spatial and temporal message contents of reverberating signals. Such a decoupling of temporal from spatial messages clarifies how a stable and precise brain code for conscious states can be generated despite the highly plastic and largely diffuse spatial functional organization of the brain. A candidate mechanism underlying such a decoupling of neural message contents is signal decorrelation, which has become an important concept in neural network theory and systems theory in general. Decorrelation reduces cross-talk between multichannel signals in complex systems such as the brain while preserving other critical signal properties. On the basis of this general assumption, the following postulates and model properties are stated.

1. Only non-conscious brain processes have enough capacity to process the complex cross-talk between spatial and temporal signals originating from various simultaneously activated and functionally specific sensory areas.
2. The temporal signatures of conscious states are generated and consolidated in reverberating interconnected neural circuits that extend across long distances well beyond functionally specific topology.
3. The activation of a temporal signature generating a conscious state depends on statistical temporal coincidence of neural activity patterns (memory representations).
4. This temporal signature is independent of signal contents or messages relative to spatial brain maps.

The circuitry generating a temporal signature would have an intrinsic and essentially arbitrary but not necessarily random topology in terms of “which cell fires first.” This intrinsic topology is solely determined by temporal resonance principles. While there is no empirically based description of resonators receiving, amplifying, and transmitting time-patterned messages in the brain, a large number of physical and biophysical phenomena can be plausibly and parsimoniously explained on the basis of resonance principles or mechanisms, as the ART simulations cited here have successfully shown. Grossberg (e.g. [31]) often invokes evolutionary pressure to explain why resonant brain codes are, indeed, likely. Here, we propose to go one step further by claiming that it is likely that evolution has produced brains capable of generating conscious states on the basis of resonant dynamics of a higher and more abstract order compared with the original resonant code of ART.

### 2.4. Functional Characteristics of the Time-Bin Model

It is likely that biological resonators, in contrast with “ordinary” resonance devices designed by humans, would have highly sophisticated operating principles, given that hundreds of functionally different kinds of cells exist in the brain. On the other hand, there is no reason why resonators in the brain would have to function with a high level of precision, provided that they operate according to some redundancy principle and the whole group of resonating cells producing a conscious state behaves in a statistically predictable way. Our model conception of temporal signal sequences forming a specific biophysical time-bin pattern that activates, maintains, and inactivates a conscious state is certainly and inevitably a simplification of reality. Such a simplification does not affect the internal validity of the model arguments stated. Their major goal is to explain how a brain system could generate conscious states through the least costly processes, on the basis of a relatively limited amount of neural resources. 

 Given the known temporal properties of conscious information processing, we suppose that conscious states may generate messages corresponding to a vast number of variable contents translated in terms of bit sequences. In the simplest possible model, any of these conscious states would be identified by a unique sequence of 1second and 0second. Thus, in the same way as bar codes provide the key to an almost infinite variety of things, these temporal sequences provide the key to conscious brain states. A given temporal code would be generated spontaneously at a given moment in early brain development then eventually be reproduced and consolidated during brain learning. Consolidation would be a result of repeated reverberation in cortical memory circuits, leading to specific resonance states which correspond to conscious states. Once a resonance circuit is formed, it is able to generate a conscious state at any given moment in time provided there is a statistically significant temporal coincidence between brain activity patterns, or memory representations. As long as this threshold of statistically significant coincidence is not attained, these memory representations in the resonant circuitry remain non-conscious or preconscious. 

**Figure 1:**
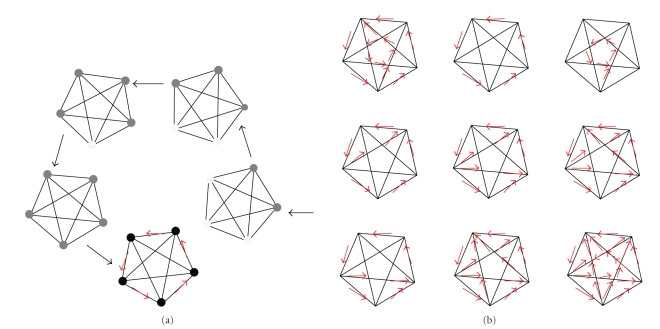
Genesis of resonance states in a dedicated circuit with five principal resonant neurons acting as “coincidence detectors.” Figure 1(a) illustrates a dedicated resonant circuit with five principal resonant neurons acting as coincidence detectors. Each apex of a given polygon corresponds to a *principal resonant neuron* which can receive input or emit output signals from and to processors anywhere in the brain, along the long-distance tracks of resonant circuitry that has been primed in the course of brain development to generate the temporal activity patterns for conscious state generation. Unidirectional priming only is shown here as one possible example, for illustration. Each edge of a polygon represents a delay path which transmits signals from a given apex to the next, with a characteristic delay that would correspond to some multiple of the elementary “bin” unit. All principal resonant neurons would have been primed throughout lifespan brain development to preferentially process input which carries statistically “strong” signals. When activated, principal resonant neurons send signals along all delay paths originating from them, and all those receiving a signal coinciding with the next input signal remain activated. The connections between principal resonant neurons of such a model would thus be potentiated as in the classic Hebbian model. Figure 1(b) shows some of the many possible excitation patterns within a dedicated resonance circuit with only five principal neurons. Such circuits would form interconnected neural networks that extend across large distances across the brain and have intrinsic, essentially arbitrary though not random, topologies in terms of “which cell fires first.” Such intrinsic topology is unrelated to functionally specific spatial cortical maps. As in the world some events are more likely than others, the same holds for brain events. Whether a given temporal resonance pattern will or will not generate a conscious state is determined by statistical likelihood computations in the brain. How such computations may work is simulated in ART (e.g., [82]), or the TEMPOTRON model by Gutig & Sompolinski [118].

 Counting from a first signal or spike in biophysical time, a temporal sequence of 1second and 0second may be described as a succession of intervals between 1's. Let us imagine a network of brain cells, or resonator, with a functional architecture or connectivity described by the shapes of closed polygons (see Figure 1 for an illustration). Each apex of such a polygon would correspond to a neuron which can receive input and emit output signals from and to processors anywhere in the brain, including along particular tracks of a resonant circuit primed for a particular temporal signature during development. Here, we refer to the apices of such a network model in terms of dedicated principal resonant neurons (PRNs). PRNs would be part of intra- and intercortical networks of neurons, capable of forming long-range connections with other neurons across large distances across the brain, well beyond their nearest neighbours, as one of their major functional property. Not all neurons in the brain would have such a capacity. 

 Each edge of a polygon would represent a delay path which transmits signals from a given apex to the next, with a characteristic delay corresponding to some multiple of the elementary “bin” unit (Δ*t*, as defined earlier by others in other models discussed earlier here). The distribution of these delays should fit the proportion of 1's and 0's in typical “time-bin” messages: if, for example, 1's are as likely to occur in a code as 0's, then the proportions of various delays Δ*t*, 2Δ*t*,…, *n*Δ*t* would be predictable. The delay paths as such would correspond to local neural architectures in the brain (e.g. [119–124]). Whatever the effective operational structure of such a resonance circuit, the specific temporal signatures it generates would be experience dependent and consolidated during development. The database of long-term memory representations from which these temporal signatures are drawn is updated continuously through non-conscious mechanisms. The conscious experience of an event we perceive as “new” is generated by a temporal resonance pattern that is for the first time activated above the coincidence threshold. Such a pattern is built from a new and unique combination of previously non-conscious memory representations. 

**Figure 2:**
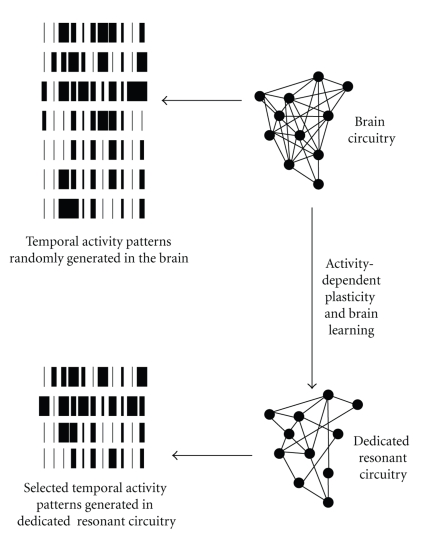
Developmental selection of temporal activity patterns coding for conscious state access. Figure 2 illustrates schematically how the critical temporal activity patterns for conscious states would be progressively selected through activity dependent plasticity during lifespan brain development. At birth, a potentially infinite number of temporal activity patterns would be generated more or less randomly in the neural circuits of the brain. As brain learning progresses, repeated matches of brain events would generate resonant states in long-distance neural circuits. Such resonant states result from higher order processing in dedicated resonant circuits which function independently from sensorial and perceptual processes. Whenever the temporal firing patterns produced by these dedicated resonance circuits reach the statistical *temporal coincidence threshold*, they generate a conscious brain state. Such a temporal code would unlock the door to consciousness like some bar code would unlock the door of an electronically protected safe.

 All PRNs would have been primed during brain development to send signals along all delay paths originating from them, and all those receiving a signal coinciding with the next input signal would remain activated. Connections between PRNs would thereby be potentiated*,* as in the classical Hebbian model. Simultaneously, signals travelling from initially activated neurons to connected cells with too long delay paths would be cancelled. Thus, once a given polygon of the resonant network is potentiated along all of its edges, it would reverberate temporally coinciding signals while amplifying resonant connections across populations of resonant neurons within massively parallel neural networks in the brain. This model assumption is biologically plausible in the light of physiological evidence for both intra- and intercortical connectivity across large distances in the primate brain. The representational power of this distributed temporal code, after functional decoupling from all spatial signal contents relating to functionally specified cortical topology, is virtually unlimited. Such a code does not imply an identity link between the spatial patterns describing a subset of PRNs (Figure 1(b)) and the temporal firing sequence recorded at any such PRN, nor is there any reason why it should. Whether the nine resonant activity patterns shown in Figure 2(b) will trigger given conscious state is not determined by the spatial activity distribution as such, but by the relative probabilistic weight or, expressed in computational language, the relative synaptic weights of the connections of a given subset of PRNs within large populations of such neurons. Thus, any of the nine different resonant states represented in Figure 2(b) would only generate a conscious state if the temporal sequence shown produces resonant activity above the statistical probability threshold. The biological plausibility of a probabilistically driven temporal code relies on the fact that, in the outside world, some physical events are more likely than others. We may consistently assume that brain events would likewise be governed by probabilistic principles. 

 Probabilistic mechanisms ensure both the relative uniqueness and the seriality of conscious brain events in a competitive race of massively distributed temporal resonances where the winner takes all. How neuronal circuits learn statistical information embedded in distributed patterns of activity is shown in some of the ART simulations by Grossberg et al. (cf. [82]). Brain learning based on purely temporal signal statistics is simulated in the TEMPOTRON model [118].

### 2.5. From Elementary Temporal Activity Patterns to a Dynamic Resonant Code

Like time-bin resonance itself, the selection of the critical temporal firing patterns that constitute the access code for conscious states would use purely statistical criteria, leading to fewer and fewer consolidated patterns for increasingly complex signal coincidences as the brain learns and develops. When we are born, all brain activity is more or less arbitrary, not necessarily random. During brain development, temporal activity patterns elicited by events in biophysical time will be linked to a variety of particular conscious experiences in a decreasingly arbitrary manner as frequently occurring codes are progressively consolidated through a process which we propose to call developmental selection. This is illustrated in Figure 2, which is our adaptation of Figure 6 from Helekar's [88] paper. Developmental selection resolves a critical problem in Helekar's theory, which fails to explain how a nonarbitrary linkage of the code to a variety of contents may take place. 

 To overcome this dilemma, Helekar daringly proposed a genetically determined linkage, which flies into the face of a large body of evidence showing that brain processes are highly plastic and experience dependent. A genetically determined linkage of the immense variety of possible subjective experience and specific temporal brain activities leaves the question of a brain mechanism for conscious states unanswered. Helekar's “elementary experience-coding temporal activity patterns” are conceived in terms of preprogrammed subsets of neural firing patterns belonging to the set of all possible temporal patterns that could be generated by the brain. His original hypothesis stated that only those patterns that are members of this subset would give rise to conscious experiences upon their repeated occurrence. The repeated occurrence of ordinary patterns, which Helekar calls noncoding patterns, would not produce conscious experience. The problem with such reasoning is that, once again, the subjective contents of a conscious state are identified with the functional nature of the state as such. In contrast to such a view, we claim that what is commonly called a “subjective experience” is encoded and decoded in the brain through non-conscious mechanisms only. 

 Also, rather than invoking a genetic programme, we prefer the far more likely hypothesis of a progressively nonarbitrary linkage of potential contents of conscious states and their temporal signatures on the basis of developmental processes and brain learning. Once a given temporal signature has been arbitrarily linked to a conscious state, it remains potentially available as a “brain hypothesis,” which is then either progressively consolidated, or not. Once consolidated, linkages between code and content may become less arbitrary, in some cases even deterministic. The progressive consolidation of linkages between code and content happens outside consciousness, through the repeated matching of working memory representations to long-term memory representations, as postulated in Grossberg's ART (e.g., [31]).

### 2.6. From Temporal Resonance to Biophysical Eigenstates

As pointed out above, what distinguishes a conscious state from a non-conscious state solely depends on a statistical threshold. A brain mechanism achieving coincidence computation would lead to the activation of a specific temporal code at a given time on the basis of statistically significant coincidences. A conscious state arises from a temporarily activated temporal signature generated within reverberating neural circuits extending across long distances in the brain. What we call “experience” in common language is coded in the brain in terms of signal sequences in biophysical time. The statistical coincidence of specific temporal activity patterns triggers, maintains, and terminates conscious brain states like a bar code would activate, maintain, and inactivate the electronic locks of a safe. Given the almost infinite number of signal sequences that are possible in a temporal code, there should be a unique temporal pattern for a unique conscious state. 

 In terms of quantum physics analogy, our time-bin resonance model suggests that non-conscious states are described by temporal wavefunctions which do not have a well-defined period. While a non-conscious state may be a combination of many nonspecific *eigenstates*, resonant activity beyond the probabilistic coincidence threshold would produce the well-defined temporal activity pattern, or wavefunction, of a single specific * eigenstate,* the “*conscious eigenstate.*” 

## 3. Arguments in Favour of the Temporal Code

The concept of a temporal neural code for conscious states as the most parsimonious link between brain and mind is justified in the light of several theoretical arguments. It might be useful here to recall that the term “code” initially stems from information theory and may stand for both (1) an entire system of information transmission or communication (like the brain) where symbols are assigned definite meanings and (2) a set of symbols for the content of a given message (like a temporal activity pattern) within that system. One argument in favour of a purely temporal access code for conscious brain states is its undeniable functional and adaptive advantage. Its origin would most likely be epigenetic. During brain development, our subjective experience remains largely non-conscious in the first months of our learning existence. Then, such experience eventually generates data of our phenomenal consciousness, around the age of two or three. 

### 3.1. Plasticity of Spatial Functional Brain Organization

Sensory, somatosensory, and proprioceptive signals may be perceived instantly as data of a conscious state, eliciting what psychophysicists call spontaneous sensations. The integration of the variety of signals such sensations originate from relies on non-conscious mechanisms, which have to be sufficiently adaptable and display a certain functional plasticity to enable the continuous updating of representations in response to changes imposed on our brains day by day by new situations and experiences. Clinical observations in neurological patients severely challenge the idea that any function should be fixed in specific *loci*. The “phantom limb” syndrome (e.g., [125, 126]) is one such example revealing the extraordinary plasticity of topological functional brain organization. The phantom limb syndrome was already mentioned in writings by Paré and Descartes, and described in greater detail by Guéniot [127]. It has been repeatedly observed in hundreds of case studies since. After arm amputation, patients often experience sensations of pain in the limb that is no longer there, and experimental data show that a third of such patients systematically refer stimulations of the face to the phantom limb, with a topographically organized map for the individual fingers of a hand. On the basis of similar evidence for massive changes in somatotopic maps after digit amputation and other experimental data showing that several years after dorsal rhizotomy in adult monkeys, a region corresponding to the hand in the cortical somatotopic map of the primate's brain is activated by stimuli delivered to the face [128], Ramachandran and his colleagues proposed their “remapping hypothesis” (e.g., [129]). The latter clarifies how spatial and topological representations are referred to other *loci* in the brain through massive cortical reorganization. The findings reported by Ramachandran and others provide compelling evidence that, despite dramatic changes in non-conscious topology, representations remain available to the conscious state and can still be experienced as sensations of pain, cold, digging, or rubbing. We believe that this is so because the higher level temporal signatures of lower level sensory representations persist for some time in the brain.

### 3.2. The Temporal “Coherence Index” and Coincidence Detection

In his “neurophysics of consciousness,” John [32, 130] suggested that a conscious state may be identified with a brain state where information is represented by levels of coherence among multiple brain regions, revealed through coherent temporal firing patterns that deviate significantly from random fluctuations. This assumption is consistent with the idea of a stable and perennial temporal code for conscious state generation despite spatial remapping or cortical reorganization. Empirical support for John's theory comes from evidence for a tight link between electroencephalographic activity in the gamma range defined by temporal firing rates between 40 and 80 Hz (i.e., the so-called “40-Hz” or “phase-locked” gamma oscillations) and conscious states (e.g. [131]). This "coherence index," with a characteristic phase locking at 40 Hz, was found to change with increasing sedation in anaesthesia, independent of the type of anaesthetic used [132]. Decreasing temporal frequencies were reported when doses of a given anaesthetic were increased. Moreover, the characteristic phase locking at 40 Hz displays coherence not only across brain regions during focussed arousal, but also during REM sleep when the subject is dreaming ([133]). Coherence disappears during dreamless, deep slow-wave sleep, which is consistent with findings reported on deeply anesthetized patients. The fact that the temporal coherence index of a conscious state is produced during focussed arousal as well as during dreaming in REM sleep phases is fully consistent with the LaBerge's idea (e.g. [21]) that dreams and conscious imagination represent functionally equivalent conscious states. Phase-locking at some critical temporal frequency may result from intracortical reverberation and may correlate with the brain mechanisms which establish arbitrary nonrandom departures from different loci or topological maps. Such maps may undergo functional re-organization. The temporal code for conscious state generation, once established, would remain intact for quite a while, keep resonating and eventually reach the critical activation threshold.

### 3.3. Adaptive Resonance Theory (ART)

Originally, Adaptive Resonance Theory was conceived as a theory of brain learning to explain how the brain generates and updates representations of continuously changing physical environments ([134]). The theory was then extended to account for related phenomena such as attention, intention, volition, and the conscious perception of visual objects (e.g., [135]) or speech (e.g., [136]). Intentions and volition lead to focus attention on potentially relevant internal or external events. These *foci* of attention lead to new representations when the brain is able to validate and integrate them into resonant states, which would include the conscious states of the brain. According to Grossberg [31], all conscious states are resonant states, triggered by external or internal events and mediated by attention or volition. ART successfully explains how the brain ensures the continuous updating of long-term memory representations through a mechanism termed top-down matching, and how repeated top-down matching can lead to resonant brain states. Since the brain is continuously confronted with all sorts of old and new events, it has to continuously generate probabilistic hypotheses to determine what all these events are most likely to be, and whether they are relevant. This involves matching working memory representations to representations stored in long-term memory. Coincidence of such bottom-up and top-down representations produces so-called matching signals, or coincidence signals which, when repeatedly generated, lead to resonant states in the brain. These are, according to Grossberg, topologically grounded in the “What” and “Where” processing streams of the brain (see [31] for an extensive review). The resonant code suggested in ART is thus tightly linked to functionally specific brain regions coding for perceptual and sensorial processes. 

 Here we argue, for reasons we have specified above, that the generation of conscious brain states is largely independent of these specific functions. It must therefore depend on a higher order and, as we suggest, purely temporal code based on resonant brain activities beyond sensorial or perceptual processes, most likely on the basis of long-distance propagation and reverberation leading to such higher level resonance. While perception and sensation may be particular aspects of a specific conscious experience (see above), the mechanisms underlying such experience are not to be confounded with the mechanisms underlying the conscious brain state as such. We suggest that the latter is accounted for at the level of an “abstract” brain process, as explained in the paragraphs dealing with the time bin model, through a biophysical code in which a single dimension (time) of neural processing is preserved. The temporal signatures for conscious state generation may be seen as an emergent property of such higher level resonant brain dynamics, which would be functionally disconnected from perceptual processes or sensations. When I fully experience *I am*, as in deep meditation, I may not perceive visually, hear sound, or experience any sensation other than total relaxation, yet, my brain is definitely in a conscious state.

## 4. Questions for the Time-Bin Model

Specific questions regarding some of the implications of the time-bin model include the following.

1. How does a conscious state arise from statistical supra-threshold activation of its temporal signature?
2. How precise would such a signature be?
3. Would it account for the generation of different levels of the conscious state in brains with different anatomical structures (brains of animals, Martians, robots)?
4. How does the biophysical time-bin code relate to variations in the subjectively experienced duration of a conscious state or psychological moment?

### 4.1. From the First Tune to a Conscious Experience in the Concert Hall

From the early days of our existence when nothing we see, feel, or do is conscious, visual, auditory, tactile, and other sensory input from multiple sources is steadily processed and progressively integrated into more and more stable memory representations through the extraordinary capacity of nonconscious brain processes. These representations progressively fill the steadily up-dated database that forms our long-term memory, from the first time we see a face or hear a tune to the moment we start recognizing tunes, pieces of music, and faces and names of performers. At some stage in this process, when there is enough resonant circuitry extending across longer and longer distances in the brain, allowing the increasingly non-arbitrary linkage between conscious states and temporal signatures capable of triggering them through coincidence statistics that have become robust enough, a greater and greater variety of non-consciously integrated representations will become available to a larger and larger variety of complex conscious experiences. When we sit in a concert hall and listen to a symphony by Brahms, we will experience successive mental events during which certain aspects of the symphony, the visual scene, or the person sitting next to us are selectively and momentarily made available to a conscious state. What is selected will depend on how many coinciding non-conscious memory representations of the sssprevious conscious states will produce activities above the threshold in the dedicated long-distance resonant circuits which generate their temporal signatures. Other brain mechanisms, such as top-down amplification or volition ([9, 31]), may or may not be involved in this process. 

 When a conscious state is triggered, we become for a short moment able to take stock of past events and to project events into the future. This ability reflects the time-ordering function of consciousness. It allows humans to plan and to read sense into their lives. Sometimes when we are conscious, we may be under the impression that what we experience looks or feels new, although we have seen or felt the same many times before. Conversely, when we find ourselves in a new situation, a conscious experience may leave us with the feeling that we have “been there before”, or that “this has happened before.” Such impressions are readily explained by the statistical nature of the temporal code proposed here.

### 4.2. Apparent Novelty and “déjà vu”

Subjective impressions of novelty or “déjà vu” would result from the fact that the temporal signatures of conscious states represent a code that is based on a purely temporal statistical likelihood. In such a code, identical signatures are not linked to identical conscious experiences. A brain hypothesis for a physical event at any given moment in time cannot be more than the brain's “best guess,” and biophysical brain events that remain identical across time do not necessarily produce identical conscious experiences across time. Conversely, conscious states with different temporal signatures may well produce a subjective experience of “*déjà vu.*” A brain code for conscious states does not have to be perfectly accurate, only sufficiently robust against major fluctuations and errors. This idea of an approximate brain code is consistent with the hypothesis of a multiple realizability of conscious states.

### 4.3. Multiple Realizability of Conscious States

Rather than assuming that there would be a unique physiological state of the brain for every unique mental state, philosophers such as Lewis (e.g., [137]) have argued that the idea of different physiological or physical life-forms being in a same mental state without being in the same physiological or physical state would be a far more plausible hypothesis. The latter has been termed “hypothesis of a multiple realizability of mental states.” Brains with different levels of physiological development and spatial functional topology or architecture should be able to generate temporal signatures producing equivalent, though not necessarily identical, conscious states in different species. This should be possible through long-distance temporal resonance in neural networks with very different intrinsic topologies and could be based on statistical activity thresholds far less robust than those established on the grounds of brain data reflecting the amount and complexity of human lifelong development. What kind of qualitative experience or *qualia* such conscious states would enable remains completely uncertain. Our conscious brain somehow becomes connected with the physical world in the course of development, through a discrete process which enables it to function in a statistically reliable way. Sometimes, this process goes wrong, as in pathological brain development producing dysfunctional conscious states.

### 4.4. The Conscious Brain and Psychological Time

**Figure 3:**
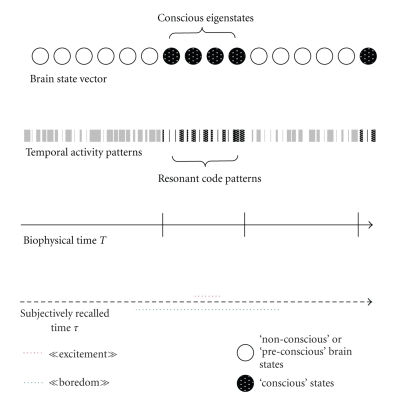
The conscious *eigenstate* as a function of biophysical and subjectively recalled time. Figure 3 illustrates how a conscious *eigenstate* of the brain may be conceived as part of a state vector as a function of biophysical time (*T*) and subjectively recalled time (**τ**). In our model, the duration of a conscious *eigenstate* would correspond to a given number of biophysical “time bins.” Biophysical time (*t*) is independent of the subjectively recalled duration of a given experience by a human individual and would correspond to the duration of the critical temporal activity pattern generated in dedicated long-distance resonant circuits to activate, maintain, and inactivate a conscious *eigenstate*. Our “time bin model” thus explains how the inner clocks of consciousness operate independently from subjective experience, where variations from “interesting” to “dull” may produce variable, subjectively recalled durations of events.

In a way similar to that of sonar systems which connect to the outside by acquiring some form of knowledge of the physical environment, conscious states appear to be encoded in our brains in terms of temporal base frequencies, as through scanning or pulsing. Although a conscious state may be experienced in any form of psychological space-time, the associated biophysical periods in the brain “scale” such experience through a completely self-sufficient code. This explains how the inner clocks of consciousness can operate independently from spatial, verbal, or any other form of cognitive or emotional experience. The brain is thus able to detach itself from the subjective nature of conscious experience, from what may seem “exciting” or “boring” to us, with time “flying by” or “standing still.” While we are in a conscious state, imprisoned by all sorts of mental events we may be experiencing, or completely freed from such experience as in fully conscious deep meditation, the brain is scaling signals related to these temporary events, in its own biophysical time (see Figure 3).

## 5. Conclusions

The abstract model of conscious state generation proposed here addresses the mind-body problem by suggesting that the conscious brain state is a dynamic result of progressive life-long brain development. The conscious state code emerging from such development is of a purely temporal and statistical nature. 

 Some time ago, Nagel [138] insisted that in order to understand the hypothesis that a mental event is a physical event, we require more than the understanding of the word “is”. His comment directly relates to identity theory (e.g., [139, 140]), a class of mind-body theories which reject dualism by considering two possibilities, or hypotheses, of identity between a mental state and a physiological state. The first is type identity, where mental states themselves would be physical states. The second is token identity, where mental states would be the direct reflection of a physiological or physical state. Our model assumptions do not sustain the identity claim. They address a fundamental problem recently discussed by Block [10]. Since phenomenal consciousness exceeds conscious cognitive activities such as perception or memory recall, a first step towards the abstract solution argued for by Block is to offer a theory that dissociates the brain origins of cognitive performance taking place within consciousness from the brain genesis of the conscious state as such. The idea of an abstract temporal signature for conscious states achieves this by explaining how a conscious state arises from higher-order temporal resonance dynamics that are independent of sensory processing and perception. 

 Thus, as Nagel [138] suggested, we go indeed beyond the word “is” when we address the mind-body problem in terms of an abstract biophysical code, which is difficult to reconcile with theories invoking “type” or “token” identity between mind and brain. We nonetheless defend a rigorously monist view by suggesting that dynamic links between conscious states and physiological states form on the basis of highly plastic brain activities governed by probabilistic principles.
